# Genome-Wide Network of lncRNA–mRNA During Ovine Oocyte Development From Germinal Vesicle to Metaphase II *in vitro*

**DOI:** 10.3389/fphys.2020.01019

**Published:** 2020-08-18

**Authors:** Jun-Jie Wang, Meng-Han Niu, Teng Zhang, Wei Shen, Hong-Guo Cao

**Affiliations:** ^1^Anhui Province Key Laboratory of Local Livestock and Poultry Genetic Resource Conservation and Bio-breeding, College of Animal Science and Technology, Anhui Agricultural University, Hefei, China; ^2^Key Laboratory of Animal Reproduction and Germplasm Enhancement in Universities of Shandong, College of Life Sciences, Qingdao Agricultural University, Qingdao, China; ^3^State Key Laboratory of Reproductive Regulation and Breeding of Grassland Livestock, College of Life Sciences, Inner Mongolia University, Hohhot, China

**Keywords:** lncRNA–mRNA network, ewe, oocytes, meiotic maturation, RNA-Seq

## Abstract

Long non-coding RNA (lncRNA) is involved in many biological processes, and it has been closely investigated. However, research into the role of lncRNA in ovine ovarian development is scant and poorly understood, particularly in relation to the molecular mechanisms of ovine oocyte maturation. In the current study, RNA sequencing was performed with germinal vesicle (GV) and in vitro matured metaphase II (MII) stage oocytes, isolated from ewes. Through the use of bioinformatic analysis, abundant candidate lncRNAs in stage-specific ovine oocytes were identified, and their *trans*- and *cis*-regulatory effects were deeply dissected using computational prediction software. Functional enrichment analysis of these lncRNAs revealed that they were involved in the regulation of many key signaling pathways during ovine oocyte development, which was reflected by their targeted genes. From this study, multiple lncRNA–mRNA networks were presumed to be involved in key signaling pathways regarding ovine oocyte maturation and meiotic resumption. In particular, one novel lncRNA (MSTRG.17927) appeared to mediate the regulation of phosphatidylinositol 3-kinase signaling (PI3K) signaling during ovine oocyte maturation. Therefore, this research offers novel insights into the molecular mechanisms underlying ovine oocyte meiotic maturation regulated by lncRNA–mRNA networks from a genome-wide perspective.

## Introduction

In mammals, fully grown follicles release mature oocytes that are ready for fertilization. Each oocyte is enclosed in a follicle and undergoes an extended period of meiotic arrest that may last 40 years or more in humans and some other animals (Woodruff et al., 2010; Conti et al., 2012), during which time the oocytes remain at the first meiotic prophase. Only when luteinizing hormones (LH) triggers ovulation in the adolescent do the germinal vesicle (GV) oocytes resume meiosis, complete the first meiosis, extrude a polar body, and then reach the metaphase of the second meiosis (MII) ready for fertilization.

A long period of meiotic prophase or GV arrest is suggested to be of great importance for the production of a fertilization-competent oocyte (Conti et al., 2012; Coticchio et al., 2013). The maintenance of this arrest involves a regulatory network between oocytes and somatic cells, thought to be dependent on high levels of cyclic AMP (cAMP) (Cho et al., 1974; Bornslaeger et al., 1986; Conti et al., 2002, 2012) that can activate protein kinase A (PKA) and contribute to the inactivation of the meiosis-promoting factor (MPF), as cAMP decreases PKA is inactivated leading to the activation of MPF, which is responsible for the resumption of oocyte development and GV break down (GVBD) (Jones, 2004; Coticchio et al., 2013). During arrest, cAMP levels within oocytes usually remain relatively high. cAMP can be specifically degraded by phosphodiesterase 3A (PDE3A); however, PDE3A activity is inhibited by cyclic GMP (cGMP) (Norris et al., 2009; Vaccari et al., 2009) that is produced by cumulus cells and diffuses through gap junctions into the oocyte to prevent initiation of meiotic resumption. Following the LH surge, the secretion of natriuretic peptide precursor type C in mural granulosa cells is prevented, which impairs cGMP synthesis (Zhang M. et al., 2010; Wang et al., 2019); as a result, PDE3A is activated, and cAMP is reduced by phosphodiesterase, which in turn permits the resumption of meiosis.

Non-coding RNAs (ncRNAs) are generally divided into two classes according to length: short/small ncRNAs [such as micro-RNAs (miRNAs)] and long non-coding RNAs (lncRNAs) with a length generally exceeding 200 bp (Nagano and Fraser, 2011; Sun and Kraus, 2013). Following the prevalence of genome-wide transcriptome research, a substantial set of roles for lncRNAs are gradually being exposed. Growing evidence suggests that lncRNAs participate in many biological processes, such as the regulation of gene expression (Kim and Sung, 2012; Rinn and Chang, 2012), cellular differentiation and development (Fatica and Bozzoni, 2014; Liu et al., 2017), apoptosis, and disease (Wapinski and Chang, 2011; Rossi and Antonangeli, 2014; Zhang et al., 2019). Moreover, lncRNAs are known to target chromatin modification complexes that mediate posttranscriptional modifications and translational control of messenger RNA (Fatica and Bozzoni, 2014); they also play a part in genomic reprogramming (Nagano et al., 2008; Loewer et al., 2010). Despite the fact that lncRNAs, such as the well-known H19 and Xist (X inactive-specific transcript), were identified as early as the 1990s (Brockdorff et al., 1991; Carninci et al., 2005), their roles are relatively poorly investigated and understood, particularly in the ovary. Presently, most studies have focused on the association of lncRNAs with ovarian cancer (Norris et al., 2009; Mitra et al., 2017), and only a few reports have investigated their roles in ovarian development and follicle growth. Battaglia et al. (2017) suggest that 41 lncRNAs interact with oocyte miRNAs and are potentially involved in the regulation of folliculogenesis in humans. Meanwhile, Liu et al. (2018) discovered that lncRNAs/transcripts of uncertain coding potential (TU) are highly expressed in goat ovaries in the luteal phase and that this might regulate the synthesis of progesterone.

RNA transcriptome sequencing is now one of the most powerful methods available for the detection and expression analysis of lncRNAs (Mortazavi et al., 2008; Atkinson et al., 2012). In the present study, we explored the lncRNA and coding gene regulatory networks of ovine oocytes from GV to MII stages. Briefly, GV oocytes that were isolated from ewes and MII oocytes matured *in vitro* were sequenced for the expression of lncRNA. Through RNA data analysis, the stage-specific lncRNAs and coding genes were identified. Furthermore, the co-expression (*trans*) and co-location (*cis*) analyses were implemented with differentially expressed lncRNAs (DELs) and genes (DEGs) to explore the regulatory networks involved in oocyte transition from GV to MII stages.

## Materials and Methods

### Ethics Statement

All the experiments in this research followed national guidelines and were in accordance with the Ethical Committee of Anhui Agricultural University. All surgery on ewes had the approval of the Institution of Animal Care and Use Committee, Anhui Agricultural University (no. 2016017), China.

### Ovine Oocyte Collection and Maturation *in vitro*

Ovine ovaries were collected from a slaughterhouse in Ürümqi in Xinjiang, China. They were then transported in saline solution supplemented with penicillin–streptomycin to the laboratory within 4 h. There, follicles of 2–8 mm in diameter were extracted from the washed ovaries. After settlement for a short time, the supernatant was removed, and the cell pellet was resuspended with washing buffer for subsequent dissection and selection under a stereoscopic microscope. The oocytes were divided into two classes: (i) denuded oocytes (GV stage) and (ii) oocytes surrounded by cumulus cells; the latter were treated with hyaluronidase (Sigma, H-3506) to remove the cumulus cells. Oocytes were then cultured *in vitro* with maturation medium in a humidified incubator at 38.5°C with 5% CO_2_ for 23–25 h. MII stage oocytes with an extruded first polar body were harvested. The sequence samples were prepared with 30–35 oocytes as one biological replicate.

As in previous reports (Peng et al., 2008; Su et al., 2014), the maturation medium (per 100 ml) for oocytes was composed of double-distilled water, 10 ml fetal bovine serum (FBS; Gibco, 00642), 0.95 g TCM199 (Gibco, 31100-03510), 0.22 g NaHCO_3_, 0.2384 g HEPES, 0.022 g Na-Py, 0.0075 g penicillin, 0.005 g streptomycin, and 0.003 g glutamine supplemented with 100 IU FSH, 1 ml 17β-E2 (working concentration is 1 μg/ml), and 200 IU LH. Phenol red was added to adjust the pH value, and the medium was filtered through a 0.22 μm filter and preserved at 4°C.

### RNA Extraction, Library Construction, and Sample Sequence

RNA samples from the collected oocytes were extracted with TRIzol reagent, and the following pipeline was performed to ensure RNA quantity: 1% agarose gel electrophoresis for RNA degradation and contamination test, NanoPhotometer^®^ spectrophotometry (IMPLEN, Westlake Village, CA, United States) for detecting RNA purity, Qubit^®^ 2.0 Fluorometer (Life Technologies, Camarillo, CA, United States) for quantifying RNA concentration, and RNA integrity assessment with an RNA Nano 6000 Assay Kit and a Bioanalyzer 2100 system (Agilent Technologies, Santa Clara, CA, United States). An aliquot of 3 μg RNA from each sample was used as the input to prepare the sequence. After removal of ribosomal RNA, the sequence libraries were generated with a NEBNext^®^ Ultra^TM^ Directional RNA Library Prep Kit (NEB, Ipswich, MA, United States) according to the manufacturer’s recommendations, the quality of which was assessed with an Agilent Bioanalyzer 2100 system. After sample clustering with a TruSeq PE Cluster Kit v3-cBot-HS (Illumina, San Diego, CA, United States) according to the manufacturer’s instructions; sequencing was conducted on an Illumina HiSeq 2000 platform using a 100 bp paired-end strategy.

### Pipeline of Data Processing and Analysis

Firstly, quantity control was performed to remove low-quality and adapter reads from the generated raw data using Trimmomatic software (v 0.38) (Bolger et al., 2014). FastQC (v0.11.8) reports were produced to check the obtained data (Andrews, 2010), which would be used for subsequent analyses. With the STAR software (v2.7.1a)^1^, clean data were aligned to the sheep reference genome (version Oar_v3.1 from ensemble) to produce Bam files. After Bam files were sorted with SAMtools (v1.9) (Li et al., 2009), transcript assembly was performed with StringTie software (v2.0) (Pertea et al., 2015). Subsequently, all samples were merged to generate one GTF file with a setting of a “-m 200” parameter, by which only transcripts with a length >200 bp were retained for the following lncRNA analyses.

### Screening for Candidate lncRNAs

**FIGURE 1:**
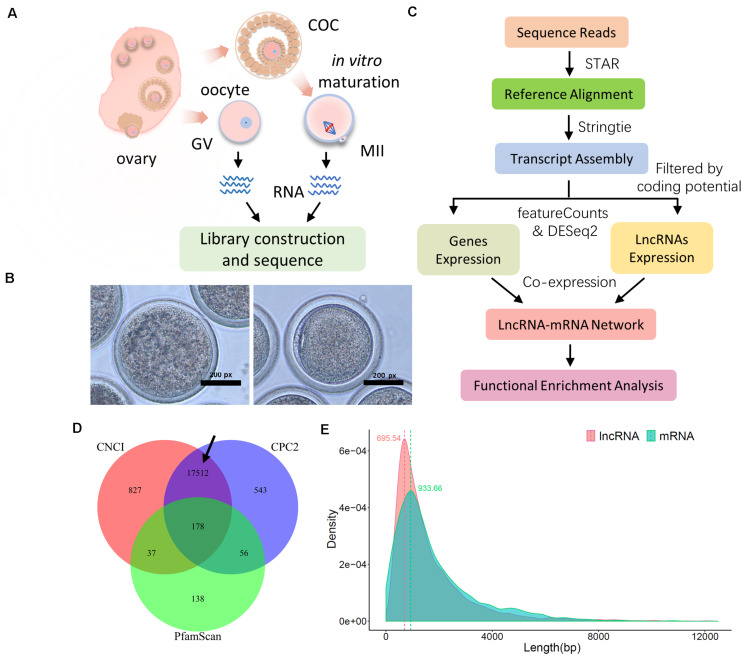
Sequence and data preprocessing of ovine oocytes at the germinal vesicle (GV) and meiosis II (MII) stages. **(A)** Schematic diagram of sample collection and RNA sequencing procedure. **(B)** Representative bright-field images of ovine GV (left) and MII (right) oocytes. **(C)** Data preprocessing pipeline for mRNA and lncRNA. **(D)** Candidate lncRNA identification with coding potential assessment of CNCI, CPC2, and PfamScan; the black arrow refers to the final candidate lncRNA. **(E)** Distribution of transcript length of lncRNA and mRNA; the transcript length of lncRNA and mRNA was generated using the assembly process of StringTie software, and density distribution was assembled using the ggplot2 package in the R environment.

We identified candidate lncRNAs to carry out downstream analyses following previously reported procedures (Gao et al., 2017; Sun et al., 2018; Zhang et al., 2019). According to the assembled transcript, preliminary filtration was executed to select non-genetic transcripts with a “classcode” label annotated by GffCompare software^2^ (v0.11.2). The produced transcript file may then contain the lncRNA transcripts with protein coding potential that should be removed. Here, we predicted the protein coding ability of lncRNA transcripts through three methods: Coding–Non-coding Index (CNCI) (Sun et al., 2013), Coding Potential Calculator (CPC) (Kang et al., 2017), and PfamScan (Finn et al., 2016). The combined analyses guaranteed the accuracy of the final candidate lncRNAs (Figure 1D, marked with black arrow) in our study.

### Expression Analysis of mRNA and lncRNA

The expression levels of mRNA and lncRNA were calculated using featureCounts (v1.6.4) (Liao et al., 2013) based on assembled transcript files. Then, the DESeq2 package (Love et al., 2014) was used to normalize expression levels and perform differentially expressed analyses for mRNAs and lncRNAs using R (v 3.6.0). In our research, *p*-values of the transcripts were adjusted using Benjamini and Hochberg’s approach for controlling the false discovery rate (FDR). Significant DEGs and DELs were determined with *p*adj (adjusted *p*-value) <0.05.

### Co-expression and Co-location Analysis

It is thought that lncRNAs play a *trans*-role through their related co-expression genes (Yan et al., 2017). In our study, the pairwise significant DEGs and DELs were estimated with Pearson’s correlation coefficient (*r*) using the R package Hmisc (v4.3)^3^. Those mRNAs with a *p*-value < 0.01 and |*r*-value| > 0.9 were considered as co-expressed genes of their lncRNAs. Further, for *cis*-regulation, based on the assembled GTF file of candidate lncRNAs, we employed FEELnc (v 0.1.1) (Wucher et al., 2017) to screen the coding genes that were located within 100 kb upstream and downstream of lncRNAs (that is, co-location), and the *cis*-regulated genes of their lncRNAs were decided with the parameter of “isBest = 1.” According to the illustration of FEELnc software, the lncRNA subtypes were divided into two classes: “Genic” and “Intergenic.” “Genic” included subtypes of “overlapping,” “containing,” and “nested”; “Intergenic” consisted of “same strand,” “convergent,” and “divergent” subtypes. Meanwhile, the core regulatory network was determined with common paired mRNA–lncRNA for co-expression and co-location modules.

### Functional Enrichment Analysis

**FIGURE 2:**
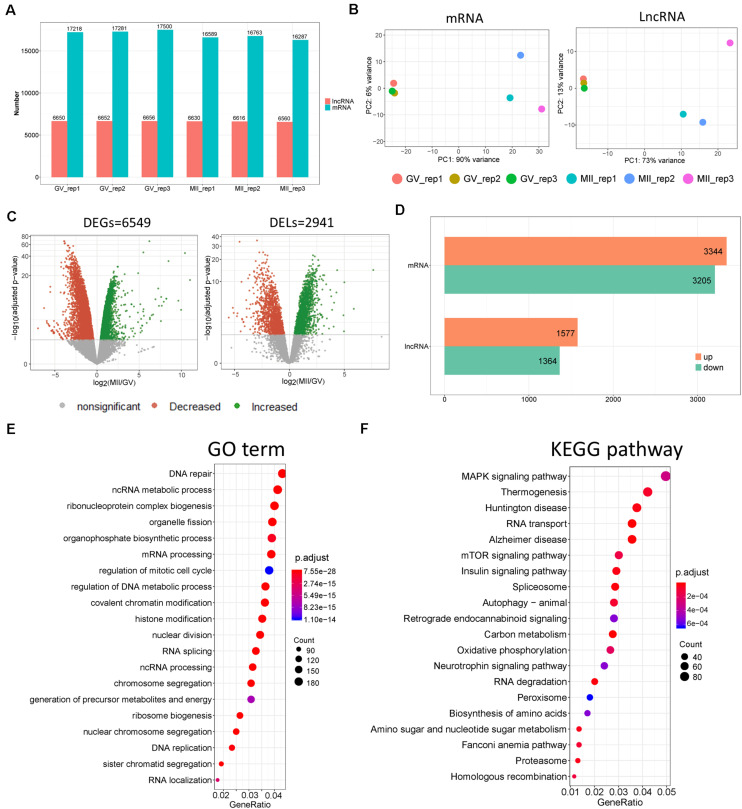
Divergent expression patterns of lncRNA and mRNA in ovine oocytes. **(A)** Number of lncRNA and mRNA transcripts with a non-zero count in each sample. **(B)** Principal component analysis (PCA) based on lncRNA and mRNA. **(C)** Volcano plots of differentially expressed genes (DEGs) and lncRNAs (DELs) of MII vs. GV stages. **(D)** Number of upregulated and downregulated DEGs and DELs of MII vs. GV stages. **(E)** GO and **(F)** KEGG enrichment of DEGs with top 20 terms; “Count” is the number of DEGs that were enriched in the term; “GeneRatio” refers to the ratio of DEG numbers that were enriched in the term vs. all DEG number.

Gene Ontology (GO) and Kyoto Encyclopedia of Genes and Genomes (KEGG) enrichment analyses were performed predominantly based on Metascape^4^ (Zhou et al., 2019), a web-based portal that operates using gene symbols as inputs and integrates a board of current biological databases. In the output results, it highlights the key and non-redundant functional terms, also the enrichment network displays intra- and inter-cluster connection of terms. Due to the limitations of Metascape, large data sets (>3.000 genes) were processed using the clusterProfiler package (Yu et al., 2012) for enrichment analysis; the top 20 most enriched terms are displayed in order according to “GeneRatio,” and the “p.adjust” value was produced using the “BH” method in the parameter of “pAdjustMethod” (Figure 2). Given the inadequate database of functional analysis in sheep, the ovine gene symbols were converted into murine homologous gene symbol IDs through the g:profile website^5^ (Raudvere et al., 2019).

## Results

### Sequence and Data Preprocessing of Ovine Oocytes at the GV and MII Stages

In the present study, denuded oocytes (GV stage) and COCs were isolated from ovine ovaries. Cumulus cells were removed from COCs and the isolated oocytes developed to the mature MII phase during a 23–25 h incubation *in vitro*. These harvested oocytes were gathered in two groups, “GV” and “MII,” for RNA sequencing (Figure 1A). Representative images of GV and MII ovine oocytes are shown in Figure 1B. To determine the molecular mechanism of lncRNA and related target genes, with quality control and high mapping rates (around 90%; Supplementary Figure S1A), the clean data were processed to identify candidate lncRNAs and coding gene transcripts according to standard pipelines (Figure 1C). In particular, candidate lncRNAs were generated after protein coding potential assessment with CNCI, CPC2, and PfamScan (Figure 1D with a black arrow). Ultimately, we obtained transcript results of lncRNAs and mRNAs with density peaks of 695.54 and 933.66 bp in length, respectively (Figure 1E), which were used for downstream analyses.

### Divergent Expression Patterns of lncRNA and mRNA

According to data processing, more transcripts of coding genes were generated than lncRNAs (Figure 2A). To understand the temporal expression patterns of lncRNA and mRNA in ovine oocyte samples, principal component analysis (PCA) was conducted (Figure 2B); it showed differing expression patterns of lncRNAs and mRNAs in GV and MII oocytes, which suggested that the mRNAs and lncRNAs displayed a temporal expression pattern in ovine oocytes dependent on the stage of development. Furthermore, volcano plots demonstrated there were 2.941 DELs (Supplementary Table S1) and 6.549 DEGs (Supplementary Table S2 and Figure 2C) of MII vs. GV stage. Moreover, the number of upregulated lncRNAs and mRNAs were both slightly more than that of downregulated ones (Figure 2D). In addition, the top 40 DELs (Supplementary Figure S1B) and DEGs (Supplementary Figure S1C) were made into a heatmap. In order to explore the biological processes of DEGs, functional enrichment was performed (Figure 2E). GO results showed that the top enriched terms were DNA repair, ncRNA metabolic processes, and ribonucleoprotein complex biogenesis; others were also closely related to cellular transcriptional states, such as mRNA processing, mitotic cell cycle, and chromatin and histone modification. Moreover, KEGG revealed that mitogen-activated protein kinase (MAPK), mammalian target of rapamycin (mTOR), and the insulin signaling pathways were likely involved in the regulation of oocyte fate during transition from GV to MII (Figure 2F).

### Co-expression Analysis of DELs and DEGs in Ovine Oocytes

**FIGURE 3:**
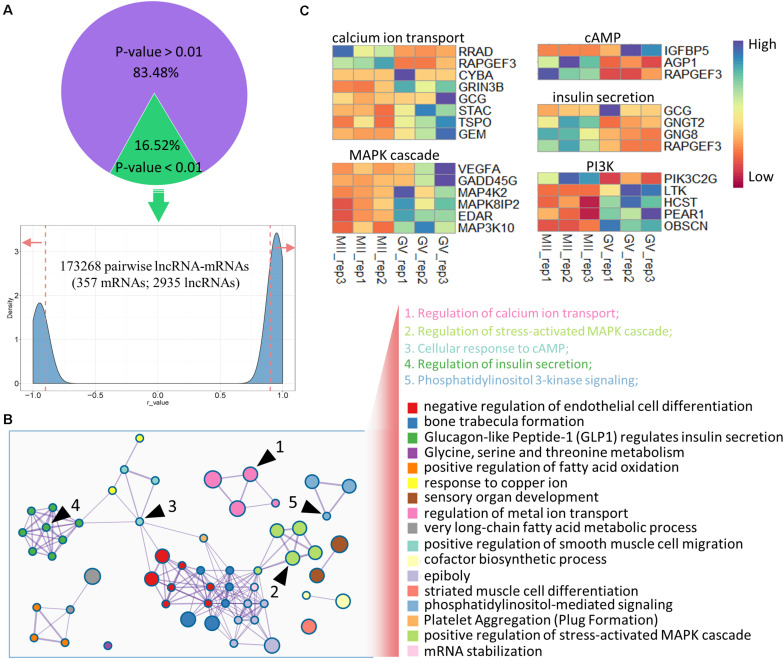
Co-expression analysis of DELs and DEGs in ovine oocytes. **(A)** Identification of pairwise lncRNA–mRNA in co-expression analysis; the dashed lines mark the *r*-value (0.9 and –0.9), and red arrows refer to the selected regions with *r*-values > 0.9 and <–0.9. **(B)** Functional enrichment analysis of co-expressed genes; similar clusters are annotated with the same color; node size represents gene numbers in term; a line of inter-clusters indicates a common gene; the five reproductive terms are marked with black arrows and labeled with numbers. **(C)** Heatmap of the expressions of genes enriched in five reproductive terms.

To precisely identify the regulatory mechanisms of lncRNAs and mRNAs, co-expression analysis was executed based on DEGs and DELs as previously reported (Liu et al., 2017; Zhang et al., 2019). After filtration based on *p*-value (*p* < 0.01, 16.52%) and Pearson’s correlation coefficient (|*r*-value| > 0.9), 173.268 pairwise lncRNA–mRNAs were produced (Supplementary Table S3), which uniquely included 357 DEGs and 2.935 DELs (Figure 3A); among these DEGs, more genes were downregulated at the MII stage (Supplementary Figure S2A). Further, GO enrichment analysis was applied using the co-expressed DEGs, and several significant GO terms were mostly connected with reproductive processes including calcium ion transport, stress-activated MAPK cascade, cellular response to cAMP, insulin secretion, and phosphatidylinositol 3-kinase signaling (PI3K; Figure 3B), despite the top terms appearing to be unrelated (Supplementary Figure S2B). In addition, the expression levels of genes enriched in the five reproductive terms are shown in the heatmap in Figure 3C, which were under the control of numerous lncRNAs (Supplementary Table S4).

### *Cis-*Regulation of lncRNAs With Target Genes

**FIGURE 4:**
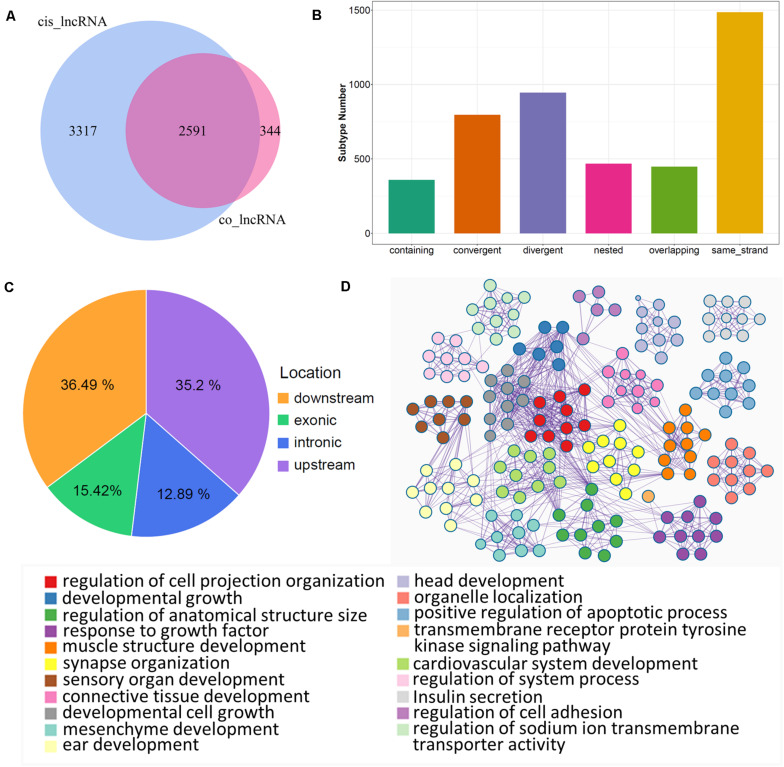
*Cis*-regulation of lncRNAs with target genes. **(A)** Venn plot of unique lncRNAs in *cis*-regulation with co-expressed lncRNAs; common region references to key *cis*-lncRNAs. **(B)** Subtype statistics of key *cis*-regulatory lncRNAs. According to the illustration, “Genic” includes the subtypes of “overlapping,” “containing,” and “nested”; “Intergenic” consists of “same strand,” “convergent,” and “divergent” subtypes. **(C)** Genome location statistics of key *cis*-regulatory lncRNAs. **(D)** Functional enrichment analysis of *cis*-genes under control of key *cis*-lncRNAs; similar clusters are annotated with the same color, node size represents gene number in term, and a line of inter-clusters indicates a common gene.

**FIGURE 5:**
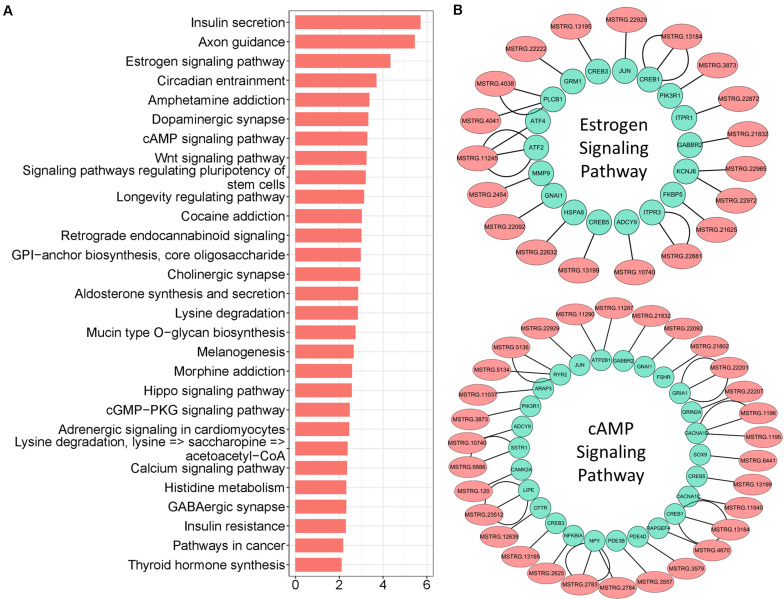
Pathway enrichment analysis of key *cis*-lncRNA targeted genes. **(A)** Pathway enrichment of key *cis*-lncRNA targeted genes. The x-axis text represent the value of “-Log(*p*-value)”. **(B)** The *cis*-regulatory networks of lncRNA–mRNA for estrogen and cAMP signaling; lncRNAs are marked with light coral nodes and targeted genes with light green; the inter-line represents targeted relationships.

lncRNAs also export a potential *cis*-regulatory capacity to their adjacent coding genes (Wang and Chang, 2011; Yan et al., 2017); therefore, FEELnc software was used to detect coding genes adjacent to candidate lncRNAs. We obtained 14.821 pairs of *cis*-regulatory lncRNA genes with the best match (Supplementary Table S5), which included 5.908 unique lncRNAs. In particular, 2.591 lncRNAs (key *cis*-lncRNAs) were common with co-expressed lncRNAs (Figure 4A), and almost half of them were increased (Supplementary Figure S3A). According to statistical analysis of the subtype, most key *cis*-lncRNAs occupied the same strand as coding genes (Figure 4B), and results of genome location showed that over one third of key *cis*-lncRNAs were located either downstream or upstream of the target gene (Figure 4C). The analysis also showed that there were 2.039 *cis*-genes under the control of these *cis*-lncRNAs. Functional enrichment suggested they gathered in cell projection organization, developmental growth, and anatomical structure size (Figure 4D and Supplementary Figure S3B). Moreover, the most enriched pathways were insulin secretion, estrogen, and cAMP signaling (Figure 5A), which are all involved in reproductive processes. Among them, the *cis*-regulatory lncRNA–mRNA networks of estrogen (Supplementary Table S6) and cAMP signaling (Supplementary Table S7) were highlighted (Figure 5B).

### Identification of Core lncRNA–mRNA Network

**FIGURE 6:**
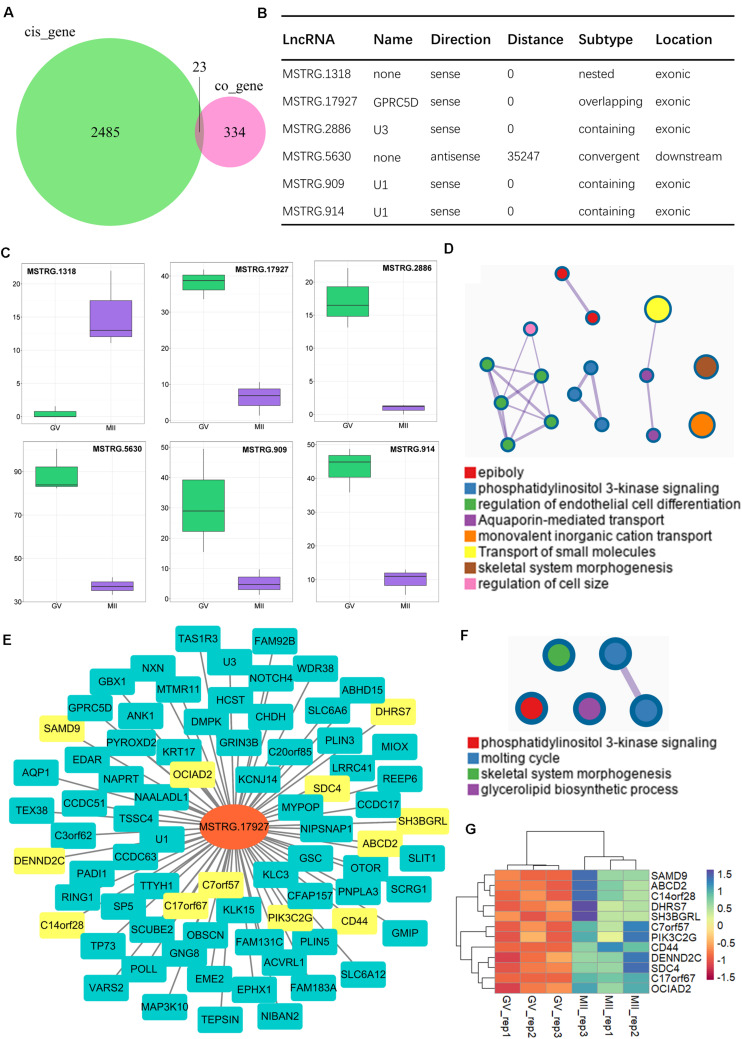
Identification of core lncRNA–mRNA networks for the maturation of ovine oocytes. **(A)** Venn plot of *cis*-regulation with co-expressed genes; common regions refer to candidate core genes. **(B)** Candidate core lncRNA–mRNA network. **(C)** Expression of core lncRNAs from GV to MII stages. **(D)** Enrichment analysis of genes targeted by core lncRNAs. **(E)** Core lncRNA–mRNA network of targeted genes and MSTEG 17927; the yellow symbol marks negative regulatory genes, and the cyan marks positive regulatory genes. **(F)** Functional enrichment analysis of targeted genes of MSTEG 17927. **(G)** Expression of negative regulatory genes targeted by MSTEG 17927.

In addition, core networks were further identified, derived from common terms of *trans*- and *cis*-regulation. In our results, 23 *cis*-genes overlapped with co-expressed genes (Figure 6A), among which six paired lncRNA–mRNA were both detected by the two methods (Figure 6B). Of note, only one lncRNA was upregulated (MSTRG.1318); others were all significantly downregulated during the GV-to-MII transition (Figure 6C). The target genes controlled by these core lncRNAs (Supplementary Table S8) mostly were enriched in epiboly and PI3K signaling, while more genes were associated with monovalent inorganic cation transport and transport of small molecules (Figure 6D). Among them, MSTRG.17927 was a candidate for tracking; there were 69 genes (which had annotated gene symbols) under its control (Figure 6E and Supplementary Table S9), which were significantly gathered in PI3K signaling (Figure 6F and Supplementary Figure S3C); among them, 12 genes increased (Figure 6G), and 57 were downregulated (Supplementary Figure S3D) during the GV-to-MII transition.

## Discussion

Long non-coding RNAs are associated with many reproductive processes in animals (Golicz et al., 2018), including sex determination, spermatogenesis, and genomic imprinting (Gabory et al., 2009; Zhang L. et al., 2010; Arun et al., 2012). However, their roles in ovarian development are poorly understood. In the present study, genome-wide candidate lncRNAs in GV and MII stage oocytes were identified; and *trans-* and *cis*-regulation of lncRNAs were inferred with their targeted coding genes based on DELs and DEGs. Moreover, lncRNA–mRNA networks were assumed to regulate key signaling pathways regarding oocyte meiotic maturation. It is important to note that the mechanisms involved in the *in vitro* maturation of oocytes are potentially different from those occurring *in vivo*. Nevertheless, the current study provides a guide to the mechanisms involved in oocyte maturation that are regulated by lncRNA.

As reported, the potential coding capability is an important index, which can be used to distinguish protein-coding genes from non-coding transcripts (Sun et al., 2013; Zhao et al., 2018). In our study, 17.512 candidate lncRNAs were identified using strict screening conditions, and only around 6.600 lncRNAs were expressed in at least one sample. It was found that the length of lncRNA transcripts was shorter than that of coding genes, consistent with many previous reports (Ran et al., 2016; Liu et al., 2018). Furthermore, the expression of lncRNA usually exerts tissue- and stage-specific patterns in animals (Liu et al., 2017; Yan et al., 2017). Thus, the abundance profiles identified here were assumed to be candidate oocyte-specific lncRNAs. Among these, we detected 2.941 lncRNAs that were differently expressed between GV and MII stage (Figure 2C); we suggest that these lncRNAs were possibly related to the specificity of the developmental stage of the ovine oocytes.

Currently, the potential functions of lncRNAs are investigated through their target genes by using *trans*- and *cis*- regulatory methods as previously described (Gao et al., 2017; Zhang et al., 2019). In this study, we demonstrated that the co-expressed genes (*trans*-regulation) might be involved in the functional regulation of calcium ion transport and cAMP response. It has been well demonstrated that calcium in many species is involved in oocyte meiotic maturation (Tosti, 2006; Miao and Williams, 2012). In addition, cAMP is responsible for oocyte meiotic arrest and resumption (Norris et al., 2009; Cao et al., 2018). In this study, *RAPGEF3*, also termed *EPAC1*, was identified as an important targeted gene of lncRNAs (Supplementary Table S4). Interestingly, *RAPGEF3* expression distinctly decreased from GV to MII stage, and the gene has been shown to serve as a cAMP-stimulated GDP exchange factor, whose mutation is known to increase the affinity for cAMP by around 2.5-fold (Dao et al., 2006). Moreover, GO enrichment analysis revealed that several co-expressed genes were enriched in MAPK and PI3K signaling, both of which are known to participate in oocyte meiotic resumption and maturation (Schmitt and Nebreda, 2002; Liang et al., 2007). In addition, lncRNA has previously been reported to be involved in cAMP and MAPK signaling pathways (Sulayman et al., 2019; Wang, 2019). Thus, we suggested that many lncRNAs might participate in oocyte maturation via *trans*-regulation.

For *cis*-regulation, the coding genes within 100 kb upstream and downstream of lncRNAs were predicted by FEELnc software. Our results indicated that key *cis*-lncRNAs mostly were enriched at the subtypes of “same strand” and “divergents”. The majority of *cis*-lncRNAs are “intergenic” lncRNAs in our study; accordingly, it suggests the intergenic lncRNAs are likely related to modified chromatin as seen in previous reports (Khalil et al., 2009; Ransohoff et al., 2018), which implies that the *cis*-lncRNA is potentially related to meiotic maturation via chromatin modification. The *cis*-regulatory effects of lncRNAs on neighboring genes have been well examined (Sun and Kraus, 2013; Yan et al., 2017). Here, with the enrichment of *cis*-regulated genes, we uncovered the *cis*-regulatory pathway of lncRNAs; more specifically, two lncRNA–mRNA networks were highlighted (estrogen and cAMP signaling). Furthermore, several *cis*-regulatory pathways of key lncRNAs were identified, which included insulin secretion, Wnt, Hippo, cGMP-PKG, and calcium signaling (Figure 5A). These results indicated that *cis*-regulatory lncRNAs likely account for a broad range of biological processes in oocytes during the GV-to-MII stage transition. Importantly, the core lncRNA–mRNA networks were identified by overlapping of the *trans*- and *cis*-methods. It is a remarkable point that the lncRNA MSTRG.17927, which declined significantly in MII stage oocytes, is thought to be involved in oocyte maturation via PI3K signaling. Matten et al. (1996) reported that the activation of a MAPK cascade is related to MPF activation, and PI3K has been suggested to be essential for insulin-induced oocyte meiotic resumption (Bagowski et al., 2001; Liang et al., 2007). Interestingly, the co-expressed genes in this study were mostly enriched in insulin secretion. It is also known that preovulatory follicles have an abundance of insulin compared with subordinate follicles in cattle (Landau et al., 2000). Insulin exposure affects meiotic chromatin during oocyte maturation and meiotic progression in mice (Acevedo et al., 2007; Das and Arur, 2017), indicating that insulin plays a role in oocyte maturation. However, lncRNA-mediated insulin secretion in oocyte maturation needs further investigation.

In summary, RNA sequencing was performed with isolated GV and *in vitro* matured MII stage oocytes. Through prediction analysis, genome-wide candidate lncRNAs that were stage-specific were identified, and their *trans*- and *cis*-regulatory functions were investigated. As a result, we suggested that multiple lncRNA–mRNA networks may be involved in key signaling pathways throughout oocyte differentiation during the GV-to-MII stage transition. In particular, MSTRG.17927 was shown to mediate the regulation of PI3K signaling. This research provides novel bioinformatic insights into the role of lncRNA in the molecular mechanisms underlying oocyte development.

## Data Availability Statement

The datasets presented in this study can be found in online repositories. The names of the repository/repositories and accession number(s) can be found below: https://www.ncbi.nlm.nih.gov/, GSE148022.

## Ethics Statement

The animal study was reviewed and approved by the Institution of Animal Care and Use Committee, Anhui Agricultural University, China.

## Author Contributions

J-JW, M-HN, and TZ conducted the experiments. J-JW analyzed the data. J-JW, H-GC, and WS wrote the manuscript. H-GC and WS designed the experiments. All authors reviewed the manuscript.

## Conflict of Interest

The authors declare that the research was conducted in the absence of any commercial or financial relationships that could be construed as a potential conflict of interest.

## Footnotes

1 https://github.com/alexdobin/STAR

2 https://github.com/gpertea/gffcompare

3 https://github.com/harrelfe/Hmisc

4 http://metascape.org/gp/index.html

5 https://biit.cs.ut.ee/gprofiler/convert

## Supplementary Material

FIGURE S1 Statistical data of RNA sequence and expression of differentially expressed genes (DEGs) and differentially expressed lncRNAs (DELs). **(A)** Table of quality control and mapping rate statistics of RNA sequences; Heatmap of top 40 DELs **(B)** and DEGs **(C)**.

FIGURE S2 Co-expression analysis of DELs and DEGs. **(A)** Heatmap of unique co-expressed DEGs; **(B)** GO enrichment analysis of unique co-expressed DEGs.

FIGURE S3 *Cis*-regulatory analysis of DELs and DEGs. **(A)** Heatmap of genes targeted by core *cis*-lncRNAs; **(B)** Functional enrichment of genes targeted by core *cis-*lncRNA; **(C)** Functional enrichment of targeted genes of MSTEG 17927; **(D)** Expression of positive regulatory genes targeted by MSTEG 17927.

TABLE S1 Results of differential expression analysis of lncRNAs.

TABLE S2 Results of differential expression analysis of mRNAs.

TABLE S3 Results of co-expression analysis.

TABLE S4 Co-expressed gene pairs of reproduction related GO terms.

TABLE S5 Classes of lncRNAs for co-location.

TABLE S6 Estrogen *cis-*gene pathway.

TABLE S7 cAMP *cis-*gene pathway.

TABLE S8 Candidate core lncRNA-mRNA networks.

TABLE S9 Target genes of MSTRG.17927.
