# Supplementary figures and images for: Genome-Wide Network of lncRNA–mRNA During Ovine Oocyte Development From Germinal Vesicle to Metaphase II in vitro
Source: Front Physiol. 2020 Aug 18;11:1019. doi: 10.3389/fphys.2020.01019 (PMC7461901; doi:10.3389/fphys.2020.01019)

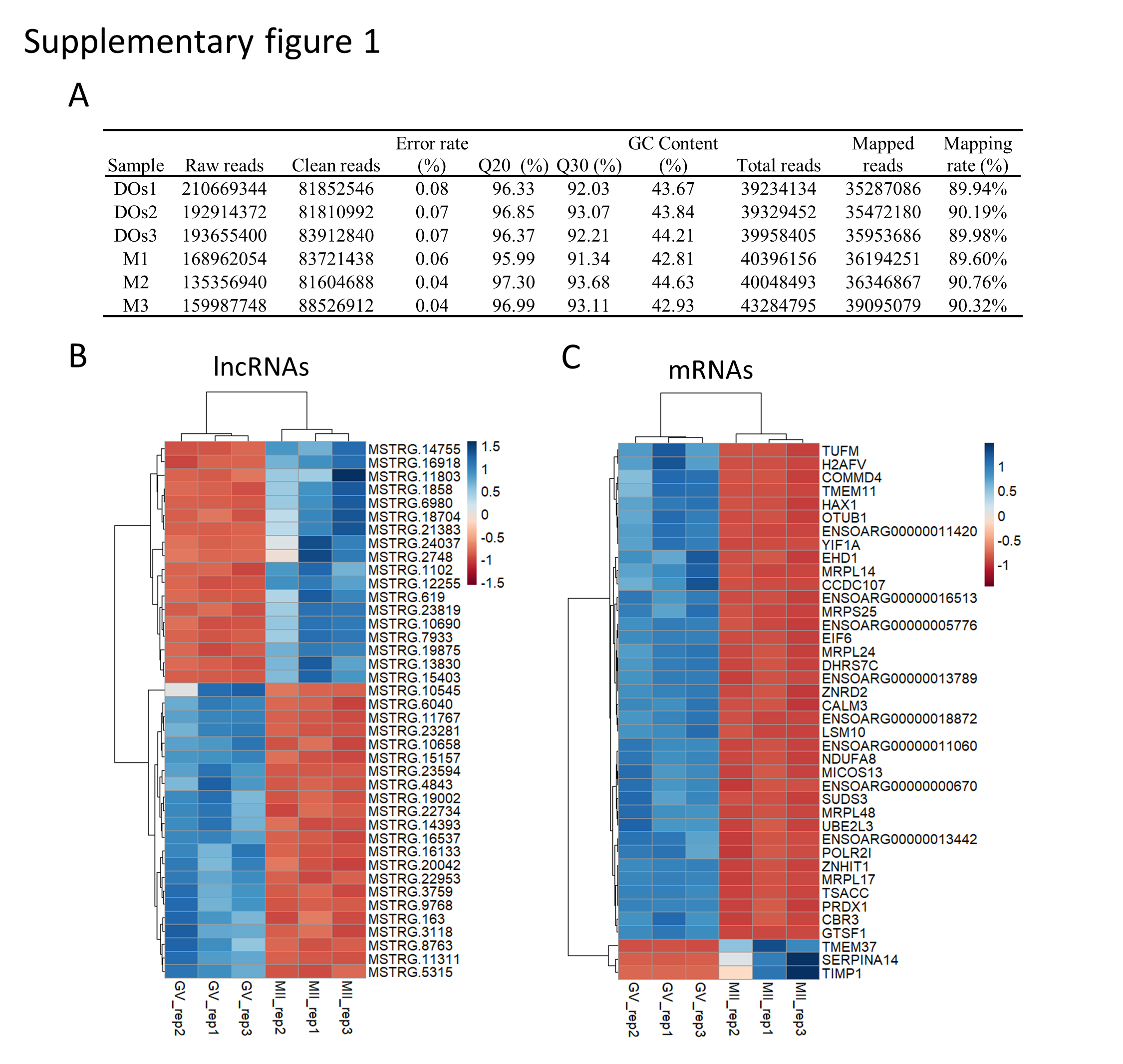

Supplement: FIGURE S1 — Statistical data of RNA sequence and expression of differentially expressed genes (DEGs) and differentially expressed lncRNAs (DELs). (A) Table of quality control and mapping rate statistics of RNA sequences; Heatmap of top 40 DELs (B) and DEGs (C). [file Image_1.TIF]

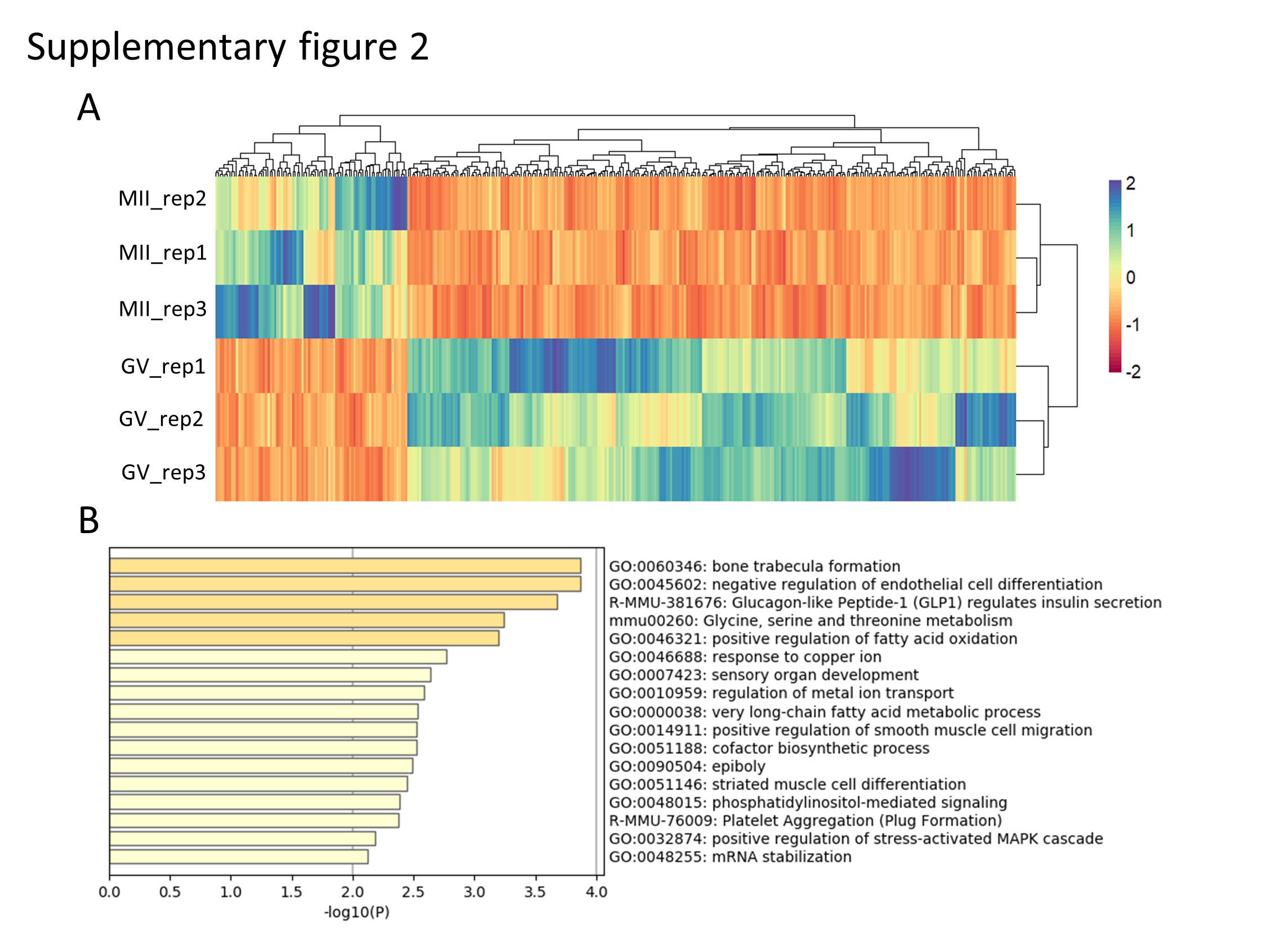

Supplement: FIGURE S2 — Co-expression analysis of DELs and DEGs. (A) Heatmap of unique co-expressed DEGs; (B) GO enrichment analysis of unique co-expressed DEGs. [file Image_2.TIF]

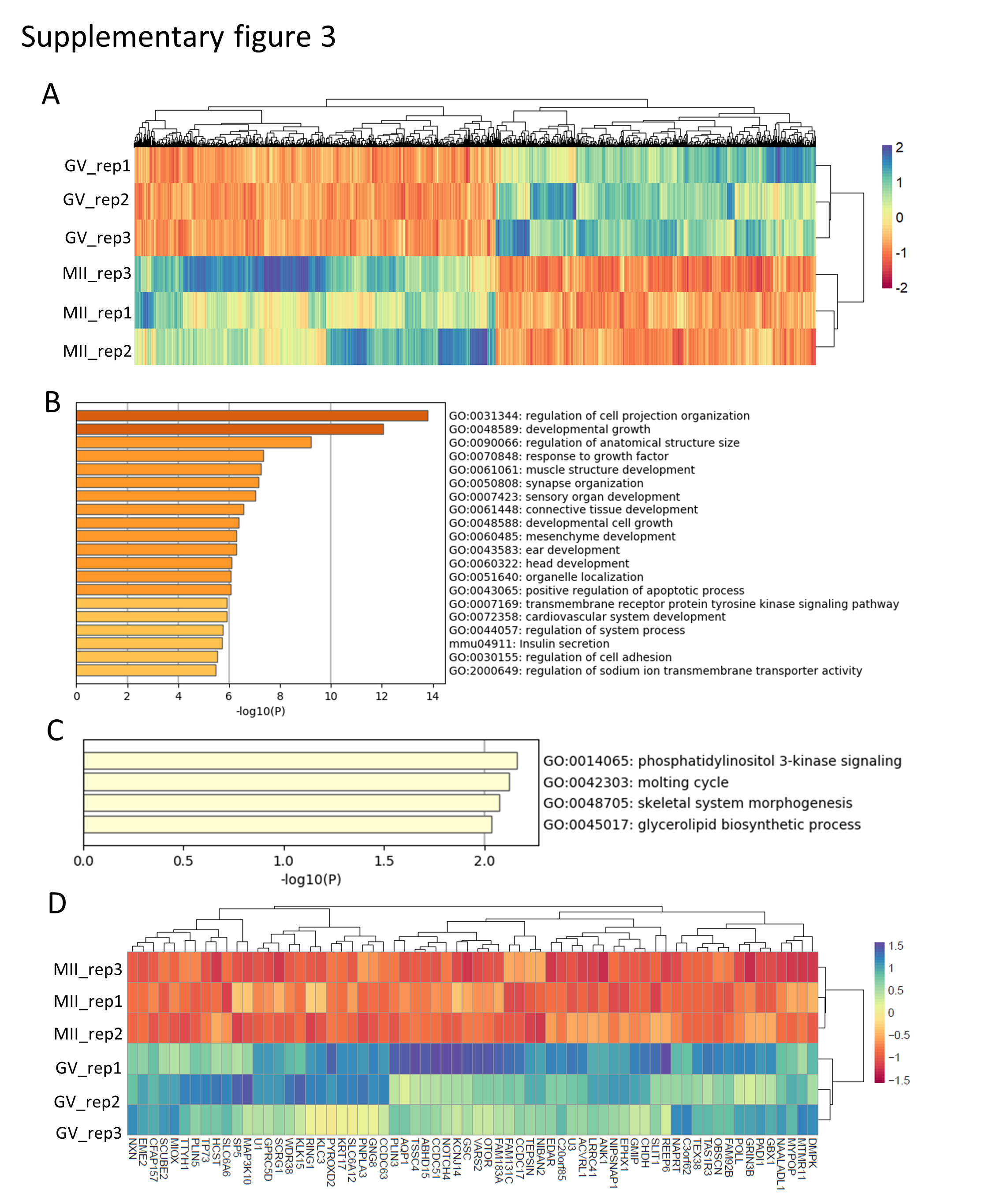

Supplement: FIGURE S3 — Cis-regulatory analysis of DELs and DEGs. (A) Heatmap of genes targeted by core cis-lncRNAs; (B) Functional enrichment of genes targeted by core cis-lncRNA; (C) Functional enrichment of targeted genes of MSTEG 17927; (D) Expression of positive regulatory genes targeted by MSTEG 17927. [file Image_3.TIF]
